# Protective Effects of Acetylation on the Pathological Reactions of the Lens Crystallins with Homocysteine Thiolactone

**DOI:** 10.1371/journal.pone.0164139

**Published:** 2016-10-05

**Authors:** Zeinab Moafian, Kazem Khoshaman, Ahmad Oryan, Boris I. Kurganov, Reza Yousefi

**Affiliations:** 1 Protein Chemistry Laboratory (PCL), Department of Biology, College of Sciences, Shiraz University, Shiraz, Iran; 2 Department of Pathology, School of Veterinary Medicine, Shiraz University, Shiraz, Iran; 3 Bach Institute of Biochemistry, Research Center of Biotechnology of the Russian Academy of Sciences, Leninsky pr. 33, Moscow, 119071, Russia; Tsinghua University School of Life Sciences, CHINA

## Abstract

Various post-translational lens crystallins modifications result in structural and functional insults, contributing to the development of lens opacity and cataract disorders. Lens crystallins are potential targets of homocysteinylation, particularly under hyperhomocysteinemia which has been indicated in various eye diseases. Since both homocysteinylation and acetylation primarily occur on protein free amino groups, we applied different spectroscopic methods and gel mobility shift analysis to examine the possible preventive role of acetylation against homocysteinylation. Lens crystallins were extensively acetylated in the presence of acetic anhydride and then subjected to homocysteinylation in the presence of homocysteine thiolactone (HCTL). Extensive acetylation of the lens crystallins results in partial structural alteration and enhancement of their stability, as well as improvement of α-crystallin chaperone-like activity. In addition, acetylation partially prevents HCTL-induced structural alteration and aggregation of lens crystallins. Also, acetylation protects against HCTL-induced loss of α-crystallin chaperone activity. Additionally, subsequent acetylation and homocysteinylation cause significant proteolytic degradation of crystallins. Therefore, further experimentation is required in order to judge effectively the preventative role of acetylation on the structural and functional insults induced by homocysteinylation of lens crystallins.

## Introduction

The cytoplasm of fiber cells in the vertebrate eye lenses is enriched with long-lived and well-ordered proteins known as crystallins [1]. These β-sheet rich and highly stable proteins are divided into α, β and γ-crystallin (Cry) which possess both structural and functional responsibilities in eye lenses [2, 3]. α-Cry is the most polymeric protein of vertebrate eye lens (about 50%) and is divided into acidic (αA) and basic (αB) subunits sharing 57% amino acid sequence homology [4]. These two protein subunits form oligomeric assemblies of various size ranges which are important in the terms of their ability to allow proper refraction of light in eye lenses. α-Cry belongs to the small heat shock proteins (sHsp) family and upon binding to the partially unfolded proteins overwhelms their aggregation [5, 6]. Therefore, the chaperone function of this protein is highly important to maintain the transparency of the eye lens over decades. Due to their limited turnover during lifespan, lens crystallins can accumulate numerous post-translational modifications lead to alterations in their structure and interactions which eventually disturb the proper refractive index of the lenticular tissues [1, 7, 8]. The complex architecture and short-range interactions among the different lens crystallins are important for the eye lens to focus light on retina. Accordingly, various modifications of these proteins interrupt their fine interactions and induce conformational change and aggregation of these proteins which eventually culminate in lens opacification and cataract [1, 7].

Lens protein homocysteinylation, which can be accelerated during hyperhomocysteinemia, is an important suspected risk factor in the development of cataract disorders. Hyperhomocysteinemia is associated with a variety of ocular diseases including retinal arteriosclerosis, glaucoma, exudative age-related macular degeneration, macular/optic atrophy, ectopic lens, age-related maculopathy and cataract [9, 10]. Under this condition, methionyl-tRNA synthetase converts a large fraction of homocysteine (Hcy) into its highly reactive cyclic thioester metabolite known as homocysteine thiolactone (HCTL). Although this conversion occurs during proofreading reaction in order to prevent incorporation of Hcy in the protein biosynthesis, protein homocysteinylation occurs in the presence of HCTL [11–13]. Genetic defects in Hcy-metabolizing enzymes (Cysteinetathionine β-synthase, methylene tetrahydrofolate reductase thiolactonase/paraoxonase and bleomycin hydrolase), vitamin B deficiency (B_6_, B_9_ and B_12_), renal impairment and high methionine intake result in a dramatic increase in the cellular levels of Hcy, leading to an increase in the synthesis of HCTL [13, 14]. The blood level of homocysteine in a healthy human subject is about 5–10 μM and increases to 15–50 μM and up to 500 μM in the mild and severe forms of hyperhomocysteinemia. The concentration of HCTL in patients with severe hyperhomocysteinemia has been reported to increase up to 59-fold and 72-fold more than the normal conditions [15]. HCTL has reactivity towards both Lys (N-homocysteinylation) and cysteine (S-homocysteinylation) residues of proteins [11, 16]. Also, HCTL has significantly higher affinity for lysine compared to cysteine residues. Protein homocysteinylation is one of the basic causes of HCTL toxicity which results in the protein structural and functional insults, leading to development of the immune responses [10, 17]. Previous studies by our group suggest that homocysteinylation of the lens crystallins results in induction of significant changes in their structural and functional characteristics. Also, the important structural alteration induced by HCTL in lens proteins has been accompanied by their increased propensity for aggregation which is important in terms of the development of lens opacity and cataract formation.

The epsilon side chain amino groups of lysine residues in proteins are known as the primary target of both acetylation and homocysteinylation [18, 19]. While homocysteinylation, which is enhanced under hyperhomocysteinemia, is pathogenic; acetylation is a physiological reaction which plays an important role in cellular regulation [11, 20]. Various acetyl transferases are responsible for the reversible acetylation of protein lysine residues by transferring the acetyl group from acetyl-CoA to the epsilon amino group of lysine [19]. Acetylation of free amino groups in proteins also occurs by acetylating medicines such as aspirin. Aspirin is a powerful anti-inflammatory, non-steroidal and anti-cataract agent whose consumption is widespread across all age groups [21]. Acetylation affects structure, stability and chaperone function of the lens crystallins [21–27]. According to previous studies, acetylation of αA- and αB-crystallins in human lenses occurs very early, even in a 15 year-old human subject and continues throughout the lifespan of the individual [23]. The *in vitro* and *in vivo* acetylation of lysine residues in lens crystallin proteins prevents their non-enzymatic glycation, and carbamylation, hence avoiding cataract formation [24, 28]. Acetylation with aspirin also inhibits the reaction of cyanate with the amino groups of lens proteins, thereby restricting human cataract development [29]. In addition, acetylation of lysine residues with acetic anhydride (Ac_2_O), prevents formation of advanced glycation end products (AGEs), improves chaperone-like activity and the anti-apoptotic properties of α-Cry [21]. Since both acetylation and homocysteinylation largely occur on lysine residues, the purpose of the current study was to take advantage of acetylation as a possible protective approach against structural and functional insults induced by homocysteinylation in lens crystallins.

## Materials and Methods

### Reagents

Homocysteine thiolactone (HCTL), 1-anilino-8-naphthalene sulfonate (ANS), thioflavin-T (ThT), dithiothreitol (DTT), 5, 5'-dithiobis-(2-nitrobenzoic acid) or DTNB, β-mercaptoethanol (β-ME), Sephacryl S-300 HR and ortho-phthaldialdehyde (OPA) were purchased from Sigma. Isopropyl-1-thio-β-D-galactopyranoside (IPTG) and kanamycin were obtained from the Merck Company. All solutions were prepared with double distilled water and kept at 4°C before use.

### Methods

#### Preparation of the bovine lens crystallins

Total soluble lens proteins (TSPs) were prepared according to our previous method [30]. In summary, the bovine eye lenses were dissected from the animals from a local slaughterhouse and after separation of the lens cortex, they were homogenized in 25 mM Tris, pH 8.0, containing 0.1 M NaCl, 0.5 mM EDTA, 0.01% NaN_3_, and 10 mM β-ME (buffer A). The solution was centrifuged at 4°C and 8000 rpm for 45 min. At the end, the supernatant was dialyzed against double distilled water (ddH_2_O) and stored at −20°C until use.

#### Expression and purification of recombinant human αA-Cry

*E*.*coli* BL21 (DE3) was transformed by a plasmid containing the αA-Cry gene (CRYAA), using an appropriate heat shock method. The transformed bacteria were grown in 800 mL of Luria Bertani broth (LB) at 37°C and shaken at 120 rpm. IPTG (0.25 mM) was added to the culture when the optical density at 600 nm of the culture reached 0.6. The incubation was continued for 5 h, then the cells were centrifuged and the pellet was suspended in three volumes of the lysis buffer (50 mM Tris pH 7.2, containing 100 mM NaCl, 1 mM EDTA, 0.01% NaN_3_ and 10 mM β-ME). After freeze-thaw, the sonication was done five times, using a Bandelin Sonopuls sonicator (Berlin, Germany). After centrifugation of the bacterial lysate at 8000 rpm for 45 min, the supernatant was filtered through a 0.2 μm filter and dialyzed against buffer B (25 mM Tris pH 6.5, containing 0.1 mM EDTA, 10 mM β-ME and 0.01% NaN_3_). To purify αA-Cry, the dialyzed samples were applied onto a DEAE-cellulose anion exchange column (2.5 cm × 13 cm) equilibrated with buffer B. αA-Cry was eluted in buffer B at a flow rate of 1 mL min^-1^ and collected from the flow through as different fractions. Appropriate fractions were collected, concentrated and further purified on a Sephacryl S-300 column (1.5 cm × 100 cm) equilibrated with buffer A. The eluted fractions with the highest protein concentration were collected and dialyzed against ddH_2_O. The purity of protein samples was confirmed by sodium dodecyl sulfate polyacrylamide gel electrophoresis (SDS-PAGE) analysis (gel 12%). At the end, the purified sample of αA-Cry was lyophilized and stored at -20°C until use.

#### The *in vitro* acetylation of lens crystallins

In the current study, Ac_2_O as acetylating agent was used to mimic the *in vivo* acetylation of lens proteins. Acetylation of these proteins was done according to the method of Nagaraj et al. with minor modifications [23]. A proper amount of Ac_2_O (500 mM in methanol) was added to solution of TSPs (5 mg mL^-1^) or αΑ-Cry (3 mg mL^-1^) in phosphate buffered saline (PBS) containing 0.01% NaN_3_, pH 7.4. The addition was done over a period of 1 h, in a drop-wise manner at room temperature. Protein/Ac_2_O molar ratios were 1:10 and 1:50 and the pH of solution was fixed at 7.4. The protein samples were finally dialyzed over night against PBS. OPA and fluorescamine assays were applied in order to determine the degree of protein acetylation. As a consequence of the reaction between free α/ε-amino groups in proteins, new adducts with particular absorption/emission characteristics are formed. Fresh OPA solution was prepared by mixing 25 mL of 0.1 M sodium borate, 2.5 mL SDS solution (20%) and 100 μL β-ME with 1 mL methanol containing 40 mg OPA. The solution volume then made up to 50 mL with ddH_2_O. A proper amount of native and acetylated proteins was incubated with 400 μL of OPA solution for 2 min at room temperature. The absorbance was then read at 340 nm with a T90^+^ UV/Vis spectrophotometer instrument (PG Instrument Ltd, London, UK).

The fluorescamine assay was also performed with a solution containing 25 μL of native and acetylated crystallin samples (1 mg mL^-1^) in 500 μL NaPi (50 mM, pH 7.4) and 225 μL ddH_2_O. The fluorescamine reagent (250 μL, 1 mM in acetonitrile) was then added to the protein solution and incubated for 10 min in the dark. The measurements were done with a Cary-100 fluorescence spectrophotometer (Varian, Australia) with the excitation/emission wavelengths were fixed at 390/490 nm [31, 32]. All measurements were repeated three times and the results reported as the average with standard deviation.

#### Protein modification with HCTL

Both native and acetylated lens proteins were incubated with HCTL (10 mM) in buffer C (100 mM NaPi containing 0.01% NaN_3_, pH 7.4) at 37°C for 3 days, with TSPs and αA-Cry at 4 mg mL^-1^ and 3 mg mL^-1^, respectively. At the end of incubation, the protein samples were individually dialyzed against buffer C in order to remove the excess reagent [33].

#### Assessment of the protein sulfhydryl with DTNB

Homocysteinylation of protein causes insertion of extra sulfhydryl groups in protein [18]. The lens crystallins (1 mg mL^-1^) were solubilized in 8 M urea, at room temperature, for overnight. The protein samples were then incubated with DTT (5 mM) for 10 min in order to reduce disulfide groups and finally dialyzed against buffer C. The evaluation of free thiol groups due to homocysteinylation of proteins was determined with DTNB (Ellman’s reagent) at a 7-fold molar excess of the protein. Then, the absorbance was measured at 412 nm, using a T90^+^ UV-Vis spectrophotometer [10]. In order to assess microenvironment of cysteine residues, the lens proteins (0.1 mg mL^-1^) were diluted in NaPi buffer (50 mM, pH 7.4). Then, DTNB was added to the solution at a 1:7 molar ratio of protein/DTNB. The kinetic profile was recorded at 412 nm in 25°C with and without SDS (2%) as a denaturant [22].

#### Fluorescence measurements

The Trp fluorescence emission spectrum of the native and modified proteins, diluted to 0.1 mg mL^-1^ in buffer C, was recorded between 300 and 500 nm with an excitation wavelength fixed at 295 nm [34]. ANS fluorescence was also performed to probe the protein's exposed hydrophobicity. The proteins (diluted to 0.15 mg mL^-1^ in buffer C) were incubated with 100 μM ANS for 30 min at room temperature in the dark. The ANS fluorescence emission was recorded between 400 and 600 nm with an excitation wavelength fixed at 365 nm [34, 35].

In addition, ThT fluorescence analysis was performed to assess the possible formation of amyloid fibrils. The protein samples (diluted to 0.15 mg mL^-1^ in buffer C) were incubated with 20 μM ThT for 5 min at room temperature in the dark. The excitation was fixed at 440 nm and the emission spectra were recorded between 450 and 600 nm [36]. All the fluorescence spectra were acquired on a Cary Eclipse spectrofluorometer (Cary-100, Australia).

#### Gel mobility shift analysis

The native and acetylated forms of total soluble lens proteins (4 mg mL^-1^) and αA-Cry (3 mg mL^-1^) were incubated with HCTL (10 mM) in buffer C at 37°C for 3 days. SDS-PAGE analysis was accomplished under both non-reducing and reducing conditions, according to the standard Laemmli protocol. 15 μg of each protein sample was loaded into wells of electrophoresis gel and visualization of the protein bands was done by an appropriate Coomassie Brilliant Blue (CBB) staining protocol [37].

#### Proteolysis of lens proteins with α-chymotrypsin

The proteolytic stability of lens proteins was investigated according to our previous method [38]. Briefly, the native and different modified forms of TSPs (2 mg mL^-1^) and αA-Cry (2 mg mL^-1^) were individually incubated with α-chymotrypsin at a ratio of 50:1 (w/w) and 100:1 of crystallin/α-chymotrypsin (w/w), respectively. The incubation was done in NaPi buffer (100 mM, pH 7.8) at 37°C for 6 h. The proteolytic reactions were stopped with boiling of the reaction mixture at 100°C for 15 min. The protein samples (15 μg in each well) were subjected to analysis with reducing SDS-PAGE (gel 12%) followed by CBB staining.

#### Circular dichroism (CD) spectroscopic analysis

The far UV-CD spectra (195–260 nm) were recorded at 25°C with a JASCO spectropolarimeter instrument (J715, USA). The protein samples were diluted to 0.3 mg mL^-1^ and the measurements were undertaken with 0.1 cm path length cuvette. All reported spectra are expressed as the molar ellipticity. Secondary structure percentages were calculated from the CD data using the DICHROWEB server with the CONTIN algorithm [39].

#### Dynamic light scattering

The dynamic light scattering (DLS) experiments were carried out using a Nanotrac Wave (Microtrac**)** dynamic light scattering instrument (USA) at 25°C**,** using laser wavelength of 780 nm and a scattering angle of 90°. The DLS data were analyzed by the Microtrac FLEX operating software [40].

#### Assessment of protein conformational stability

Conformational stabilities of the native and acetylated TSPs and αA-crystallin were determined with individual incubation of each protein (0.1 mg mL^-1^ diluted in NaPi buffer 50 mM, pH 7.4) in the presence of increasing concentrations of urea (0–8 M) at room temperature overnight. Trp fluorescence spectra were recorded between 300 and 500 nm, using an excitation wavelength of 295 nm. To determine the denaturation process, the fluorescence intensity ratios of I_350_/I_335_ for different protein samples were obtained and plotted against increasing urea concentration. In order to quantify the stability parameters, all denaturation profiles were analyzed with a three-state fitting procedure, according to the following equation:

$$F=\frac{F_{N}\;+\;F_{I}\text{exp}\left(-△G_{1}^{0}\;+\;m_{1}\left[Urea\right]\right)/RT\;+\;F_{U}\text{exp}\left(-\;△G_{2}^{0}\;+\;m_{2}\left[Urea\right]\right)/RT}{1\;+\;exp\left(-△G_{1}^{0}\;+\;m_{1}\left[Urea\right]\right)/RT\;+\;exp\left(-△G_{2}^{0}\;+\;m_{2}\left[Urea\right]\right)/RT}$$ (1)

where F_N_, F_I_ and F_U_ indicate the fluorescence ratios I_350_/I_335_ for folded, intermediate and unfolded states of each protein, respectively. ΔG^0^_1_ represents the standard free energy changes between the native and intermediate states, while ΔG^0^_2_ represents the standard free energy changes between the intermediate and unfolded states. ΔG^0^ (the sum of ΔG^0^_1_ and ΔG^0^_2_) is the free energy change between the folded and unfolded states (free energy changes of unfolding) in the absence of urea. The transition midpoint (C_1/2_) and ΔG^0^ values (kJ mol^−1^) for the native and acetylated proteins were assessed [22, 23].

#### Assessments of the chaperone-like activity

The molecular chaperone-like activity of αA-Cry (0.1 mg mL^-1^) was assessed in NaPi buffer (50 mM, pH 7.4) with the bovine pancreatic insulin (0.3 mg mL^-1^) as the target protein. Development of insulin aggregation due to addition of DTT (20 mM) was recorded at 360 nm with a T90^+^ UV-Vis spectrophotometer (PG Instrument Ltd, UK) equipped with a Peltier temperature controller (Model PCT-2). Also, the chaperone-like activity in terms of percentage of protection was quantified with the following equation:

$$\%Protection=\left(1-\frac{Ar}{Ar_{0}}\right)\times 100$$ (2)

In this equation, Ar and Ar_0_ are the area under the curves of optical density versus time (t = 20 min) in the presence and absence of chaperone, respectively [38, 41].

#### Scanning electron microscopy study

Scanning ultramicrographs of the lens crystallin aggregates were acquired with acceleration voltages of 20 and 30 kV. Protein solution (1 μL, 2 mg mL^-1^) was placed on a glass lamel and dried in air for 20 min. Subsequently, the protein specimens were coated with gold, using a Desk Sputter Coater (Dsr1 Nanostructural coating) and observed via a scanning electron microscope (SEM) (TESCAN Vega 3).

#### Protein assay

The concentration of protein was specified by UV absorption at 280 nm. An extinction coefficient of 0.72 (mL mg^-1^cm^-1^) was used for αA-Cry, as has previously been described [42].

#### Statistical analysis

The results were statistically analyzed by a one-way ANOVA test, using Sigma Plot 12.0 software. Statistical significances among the different groups were determined, using an analysis of variance, with 0.05 considered as significant.

## Results

### *In vitro* acetylation of the lens crystallins

**Fig 1:**
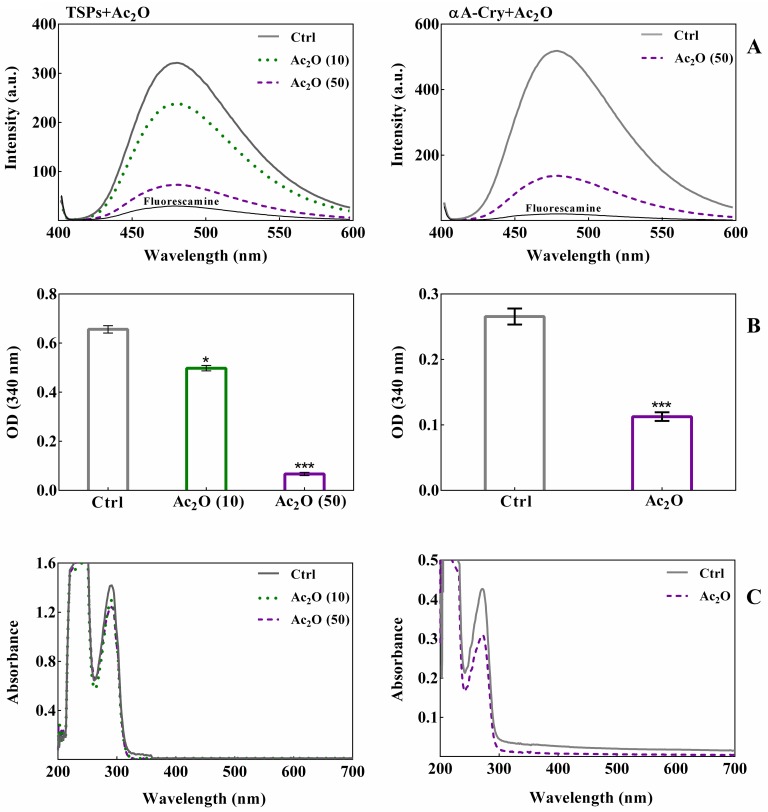
The *in vitro* acetylation of TSPs and recombinant αA-Cry with Ac_2_O. TSPs (5 mg mL^-1^) and αA-Cry (3 mg mL^-1^) were incubated respectively with different molar ratios of Ac_2_O at room temperature over 1 h. Panel (**A**) Fluorescamine assay was performed at excitation/emission wavelengths of 390/490 nm as described in the experimental section. The excitation/emission slit widths were set at 10 nm for all protein samples. Panel (**B**) The spectroscopic evaluation of protein acetylation was performed via an OPA assay. Native and acetylated TSPs (4 mg mL^-1^) and αA-Cry (3 mg mL^-1^) were added to OPA solution and incubated for 2 min at room temperature. Then, the absorbance was measured at 340 nm. All results are reported as the average of triplicate experiments. The bars represent the means ± standard deviation (SD) of three independent experiments. The values of *p < 0.05 were considered significant. Panel (**C**) UV-Vis spectroscopic analysis of acetylated TSPs and αA-Cry with Ac_2_O. All protein samples were diluted to 0.5 mg mL^-1^ prior to collect the absorption spectra between 200 and 700 nm. Ac_2_O (10) and Ac_2_O (50) indicate protein to Ac_2_O molar ratios of 1:10 and 1:50, respectively.

Acetylation of the lens crystallins causes a significant decrease in the levels of free epsilon amino groups [29]. In order to assess the acetylation upon incubation of these proteins with Ac_2_O, both fluorescamine and OPA assays were performed. The results of fluorescamine assay as shown in Fig 1A suggest that acetylation prevents the reaction of amino groups with fluorescamine, resulting in significant reduction of fluorescence intensity of the dye. Thus both the total soluble lens proteins and recombinant human αA-Cry are highly vulnerable to extensive acetylation with Ac_2_O. These results were confirmed with OPA assay as indicated in Fig 1B.

A marked reduction in the OPA absorption spectra of the lens proteins incubated with Ac_2_O suggests their extensive acetylation. To examine the structural changes upon acetylation, the absorption spectra of lens proteins were monitored between 200 and 700 nm (Fig 1B). The maximum absorption centered at 280 nm reflects both number and microenvironment of the aromatic amino acid residues and their alteration as a result of modification. Enhanced intensity of UV absorption at 280 nm implies the exposure of aromatic residues to the solvent, while reduction is linked to their burring into the hydrophobic core of the protein [43, 44]. According to Fig 1C, the reduction in absorption intensity around 280 nm after acetylation of lens proteins may indicate greater protein compactness. In addition, the increment of absorbance in the visible region of the unmodified crystallins provides an important sign for protein aggregation. Overall, while the acetylated proteins are resistant to aggregation; the non-acetylated protein counterparts demonstrate a propensity for aggregation.

### Gel mobility shift analysis of the lens crystallins upon acetylation with Ac_2_O

**Fig 2:**
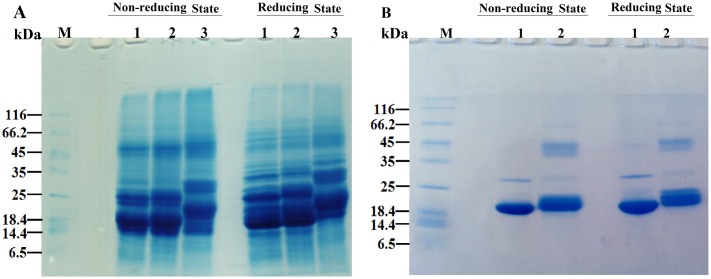
SDS–PAGE analysis of acetylated TSPs and αA-Cry with Ac_2_O and ASA. TSPs (4 mg mL^-1^) and αA-Cry (3 mg mL^-1^) were acetylated with Ac_2_O at room temperature over 1h. Then, a 15 μg of each protein sample was subjected to the reducing and non-reducing SDS-PAGE analysis (12% gel). (**A**) The SDS-PAGE analysis of TSPs acetylation. Lanes 1, 2 and 3 indicate native, acetylated TSPs which incubated with 1:10 and 1:50 molar ratios of TSPs/Ac_2_O, respectively. (**B**) The SDS-PAGE analysis of αA-Cry acetylation. Lanes 1 and 2 stand for native and acetylated αA-Cry with 1:50 molar ratio of αA-Cry/Ac_2_O. The staining of protein bands was done with an appropriate Coomassie Brilliant Blue (CBB) staining method. Ctrl and M indicate control sample and molecular mass markers, respectively.

SDS-PAGE analysis was undertaken to assess the oligomerization of lens crystallins upon incubation with Ac_2_O (Fig 2). As shown, lens proteins in the presence of Ac_2_O indicate a shift-up in the bands corresponding to the native proteins.

Shifting the protein bands in SDS-PAGE gel provides a further indication for acetylation of these proteins. As the concentration of this modifying agent (Ac_2_O) increases, the corresponding protein bands move further. Comparison between the results of reducing and non-reducing gels, suggests that acetylation may induce, to some extent, disulfide protein crosslinking. Although the extent of disulfide crosslinking accompanied with protein acetylation to somehow is very low; this type of protein crosslinking is important because it has been already indicated in the pathogenesis of cataract disorders [45].

### Assessment of conformational stability of the lens crystallins upon acetylation

**Fig 3:**
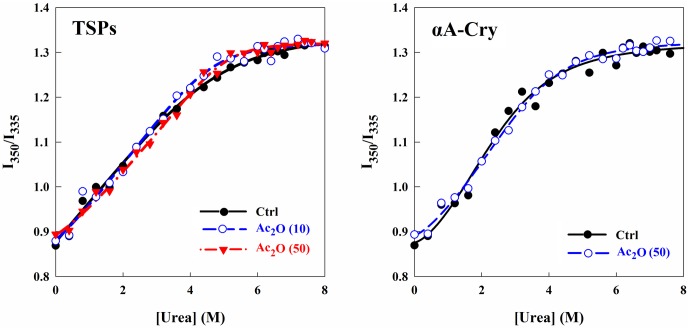
Estimation of the structural stability of lens proteins upon acetylation. The protein samples were incubated in the presence of increasing concentrations of urea (0–8 M) for 12 h in NaPi buffer (50 mM, pH 7.4). At the end of incubation, Trp fluorescence emission spectra were acquired between 300–500 nm. The results are presented as the ratio of fluorescence intensities at 350 and 335 nm for different protein samples against urea concentrations.

Due to the remarkably limited turnover of lens crystallins during life span, their stability is an important consideration. In order to determine the effect of acetylation on the structural stability of lens crystallins, chemical unfolding was utilized (Fig 3) via the addition of urea and was monitored as a function of Trp fluorescence intensity. Intensity values at 350 and 335 nm indicate the maximum intensities for completely unfolded and fully folded states of these proteins, respectively. The denaturation profiles were extracted from the sigmoidal unfolding curves according to Eq 1, and the data are presented as Table 1.

**Table 1:** Free energy and transition midpoint of urea denaturation of native and acetylated TSPs and αA-Cry.

Protein	ΔG^0^ (kJ mol^-1^)	[Urea]_1/2_ (M)
**TSPs**	14.63 ± 0.92	3.16 ± 0.57
**TSPs+Ac**_**2**_**O (10)**	14.75 ± 0.89	3.65 ± 0.43
**TSPs+Ac**_**2**_**O (50)**	15.02 ± 1.12	3.50 ± 0.73
**αA-Cry**	15.46 ± 0.95	3.64 ± 0.62
**αA-Cry+Ac**_**2**_**O (50)**	14.87 ± 1.20	3.52 ± 0.56

Ac_2_O (10) and Ac_2_O (50) stand for protein to Ac_2_O molar ratios of 1:10 and 1:50, respectively.

The transition midpoint (C_1/2_) is specified as the urea denaturant concentration at which both folded and unfolded states of the protein are equally existed in the environment [46]. The transition midpoint for the native crystallins was 3.16 ± 0.57 M. The transition midpoints of the acetylated crystallins incubated with 1:10 and 1:50 molar ratios of protein to Ac_2_O were 3.65 ± 0.43 and 3.50 ± 0.73 M, respectively. Moreover, the ΔG^0^ of chemical unfolding for the native lens crystallins was 14.63 ± 0.92 kJ mol^-1^ and the ΔG^0^ values for the acetylated crystallins incubated at protein to Ac_2_O ratios of 1:10 and 1:50 were 14.75 ± 0.89 and 15.02 ± 1.12 kJ mol^-1^, respectively. Overall, acetylation had no significant effect on the chemical stability of lens soluble proteins. On the other hand, the transition midpoint for the acetylated αA-Cry (3.52 ± 0.56 M) was not changed compared to its native protein counterpart (3.64 ± 0.62 M). In addition, the ΔG^0^ values of chemical unfolding for the native and acetylated αA-crystallins were 15.46 ± 0.95 and 14.87 ± 1.20 kJ mol^-1^, respectively. Our data suggest that acetylation of αA-Cry has little effect on its chemical stability.

### Acetylated lens crystallins partially resists against homocysteinylation

**Fig 4:**
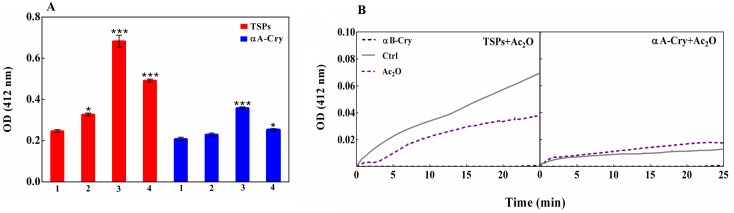
The assessment of sulfhydryl groups in lens proteins. (**A**) Evaluation of free sulfhydryl groups via DTNB assay. Native and modified TSPs (1 mg mL^-1^) and αA-Cry (1 mg mL^-1^) were solubilized with urea (8M) and then incubated with DTT (5 mM). Subsequently, the protein samples were dialyzed against buffer C. Evaluation of free thiols was determined with DTNB at a 7-fold molar excess of the protein. The absorbance was measured at 412 nm. All results are reported as the average of triplicate experiments. αB-crystallin was used as negative control because this protein has no Cys residue. The bars represent the means ± standard deviation (SD) of three independent experiments. The values of *p < 0.05 and ***p < 0.001 were considered significant. Also, 1, 2, 3 and 4 stand for control, acetylated, homocysteinylated and double-modified protein samples, respectively. (**B**) Kinetic analysis of the reaction of DNTB with thiol groups of lens proteins. The kinetic profiles of DTNB reaction with native and acetylated proteins (0.1 mg mL^-1^) were measured over 25 min in NaPi buffer (50 mM pH 7.4), containing EDTA (1 mM) at 25°C. Protein/DTNB was added at a molar ratio of 1:7 and the absorbance was measured at 412 nm.

Lysine residues are the prime target of protein N-homocysteinylation with HCTL. This cyclic thioester can also modify cysteine residues to a lesser extent, via S-homocysteinylation. HCTL and Ac_2_O could compete for modification of protein lysine residues. Therefore, in this study the lens proteins were acetylated first with Ac_2_O and then incubated with HCTL in order to evaluate the possible protection of acetylation against homocysteinylation which is known as a pathologic reaction. N-homocysteinylation results in the incorporation of additional thiol groups into a protein. The number of free sulfhydryls was measured with Elman’s reagent (Fig 4A). This experiment was performed by incubation of the native and acetylated forms of the total soluble lens crystallins and human αA-Cry with HCTL. The results as indicated in Fig 4A are average of the triplicate independent measurements.

The native lens proteins which had been incubated with HCTL displayed a significant increase in their thiol content. The acetylated proteins (TSPs and αA-crystallin) that were treated with HCTL showed notably lower quantity of thiol groups compared to the untreated proteins incubated with this reactive amino acid derivative. This observation suggests that acetylation can partially prevent the modification of lysine residues via N-homocysteinylation. The reaction of free thiols with DTNB was also followed in a kinetic fashion to mimic their reaction with HCTL via S-homocysteinylation. This experiment provides useful information on the microenvironment and reactivity of the protein thiol groups.

Based on the slope of kinetic curves (Fig 4B), the thiol groups of total soluble lens proteins showed significantly higher reactivity with DTNB than their acetylated counterparts. Our results suggest that acetylation limits the reaction of thiol groups of lens proteins with DTNB. As the experiment was repeated for untreated and acetylated αA-Cry, their thiol groups indicated approximately similar reactivity with DTNB. This observation might be a consequence of variation in the number of cysteine residues between αA-Cry and other crystallin subunits of the lens. As reported earlier, while αA-Cry contains only two cysteine residues, γ- and β-crystallins have a larger number of cysteine in their primary structures. For example γC-Cry and βB2-Cry contain 10 and 13 cysteine residues respectively [10, 32].

### The acetylated crystallins have decreased aggregation propensity upon incubation with HCTL

**Fig 5:**
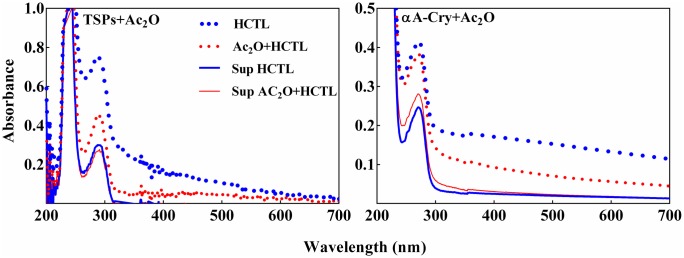
Aggregation propensity of acetylated TSPs and αA-Cry in the presence of HCTL. The native and acetylated TSPs (4 mg mL^-1^) were incubated with HCTL (10 mM) in buffer C at 37°C for 3 days. Then, the protein samples were diluted to 0.5 mg mL ^-1^ in the same buffer and the absorption spectra were collected between 200 and 700 nm. Also, the protein samples were centrifuged at 13,000 rpm for 30 min. The absorption spectra of supernatant were obtained over the same wavelength range. The symbols used in this figure are thick blue dotted line: HCTL-modified proteins; thin red dotted line: double-modified protein; thick blue solid line: supernatant of HCTL-modified protein; thin red solid line: supernatant of double-modified proteins.

Various modifications due to UV light, oxidation, glycation, crosslinking and proteolysis can affect the chaperone-like activity of αA-Cry and its ability to prevent the aggregation of target proteins. These types of modifications lead to aggregation of other lens crystallins which eventually culminates in the development of lens opacity and cataract development [1, 5, 6]. In this study, both native and acetylated lens proteins (4 mg mL^-1^) were incubated with HCTL for three days at 37°C. The samples were then diluted to 0.5 mg mL^-1^ and their absorption spectra were collected (Fig 5). The protein samples were also subjected to centrifugation at 13000 rpm, for 30 min. The significant increase in the absorbance of native proteins incubated with HCTL and their precipitation upon centrifugation provide indication of protein aggregation. Also, as indicated in Fig 5, the acetylated protein samples had significant resistance against HCTL-induced aggregation.

The soluble proteins in the supernatant may have either little or no HCTL modification. Therefore, the optical density at 280 nm of the supernatant of HCTL-modified sample was subtracted from those of the native protein counterparts to indicate the percentage of protein precipitation after modification with HCTL. Accordingly, the amount of precipitation for the native and acetylated proteins incubated with HCTL was 55% and 28%, respectively. In addition, the quantity of precipitation of native and acetylated αA-Cry incubated with HCTL was 46% and 30%, respectively. Overall, acetylation significantly reduces crystallin proteins precipitation induced with HCTL.

### Assessment of structural changes of the lens crystallins upon modification with Ac_2_O and HCTL

**Fig 6:**
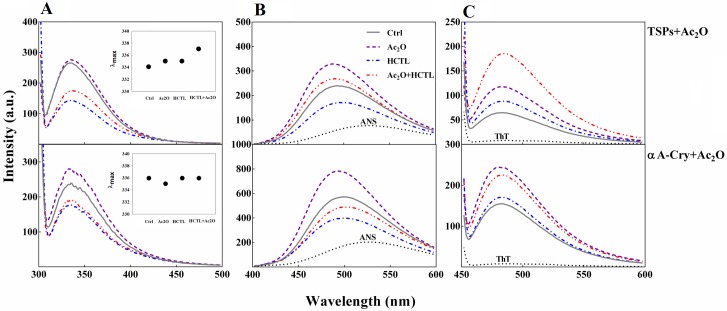
Fluorescence analysis of lens proteins upon modification with Ac_2_O and HCTL. (**A**) Trp fluorescence assessment of lens proteins. Protein samples including native and acetylated proteins incubated without and with HCTL diluted to 0.15 mg mL^-1^ in buffer C. The excitation wavelength was 295 nm and emission spectra were collected between 300 and 500 nm. The excitation/emission slit widths were 5/10 nm for TSPs and 10/10 nm for αA-Cry samples. The inset figures display λ_max_ for control (334 nm), acetylated (335 nm), homocysteinylated (335 nm) and doubled modified (337 nm) TSPs. Also, this value for the control sample, homocysteinylated and doubled modified αA-Cry is 336 nm and for acetylated αA-Cry is 335 nm. (**B**) ANS fluorescence analysis of the lens proteins. Protein samples (0.15 mg mL^-1^ diluted in buffer C) were incubated with ANS (100 μM) for 30 min. The ANS fluorescence emission spectra were collected between 400 and 600 nm, with an excitation wavelength of 365 nm. The excitation/emission slit widths were set at 10/10 nm for TSPs and at 10/20 for αA-Cry. (**C**) The fluorescence experiment was performed with incubation of the protein samples (0.15 mg mL ^-1^ diluted in buffer C) in the presence of ThT (20 μM) for 10 min. The protein samples were excited at 440 nm and the slit widths for excitation/emission were fixed at 10/10 nm.

Tryptophan fluorescence has extensively been used to study protein conformational changes, due to the sensitivity of both emission wavelength and intensity to the local environment of the indole chromophore [47]. As shown in Fig 6A, the Trp fluorescence intensity of the total soluble lens proteins slightly increased upon acetylation and there was a significant reduction after homocysteinylation. These observations suggest that polarity of local Trp is altered in different directions when these proteins are subjected to different types of modification. Similar results were obtained when αA-Cry was treated with these modifying agents. When the acetylated proteins were treated with HCTL, the Trp fluorescence spectra of double modified proteins had properties in between those in the presence of each modifying agent. Also, as indicated in the inset figures, λ_max_ for acetylated and homocysteinylated TSPs (335 nm) is nearly close to that of the control protein sample (334 nm). However, this value slightly increased in the double modified lens crystallins (337 nm). As a result of modification of αA-Cry with either Ac_2_O or HCTL, this value remains almost unchanged. However, the strong sensitivity of Trp fluorescence intensity to the protein microenvironment is routinely exploited to follow a variety of protein changes, e.g., ligand/substrate binding and folding/unfolding [48]. Reduction in fluorescence intensity seems to be due to the structural alteration of these proteins upon the above mentioned modifications.

The extrinsic fluorescence monitored by ANS dye (Fig 6B) revealed that while acetylation enhanced the hydrophobicity of total lens proteins and αA-Cry, incubation of these proteins with HCTL reduced their solvent exposed hydrophobic surfaces. The fluorescence emission of the double-modified lens proteins indicated an intermediate intensity similar to the control proteins. Overall, the results of both Trp and ANS fluorescence assessments suggest a possible shielding effect of acetylation against structural insults induced by HCTL to the lens proteins.

Several works have previously been published suggesting that HCTL can induce protein fibrillation [49–51]. The impact of different modifications on the lens proteins fibrillogenesis was examined by ThT fluorescence. As indicated in Fig 6C, ThT fluorescence intensities were significantly enhanced upon modification of the lens crystallins with the acetylating agent. The double modified protein samples were also indicated an important increment in ThT fluorescence intensity. In addition, when these proteins were incubated with HCTL, only a slight increment in ThT fluorescence emission was observed. The increased ThT fluorescence emission may suggest the formation of amyloid fibril formation by lens proteins upon different modifications.

### The impact of acetylation and homocysteinylation on the secondary structural contents and proteolytic stability of the lens crystallins

**Fig 7:**
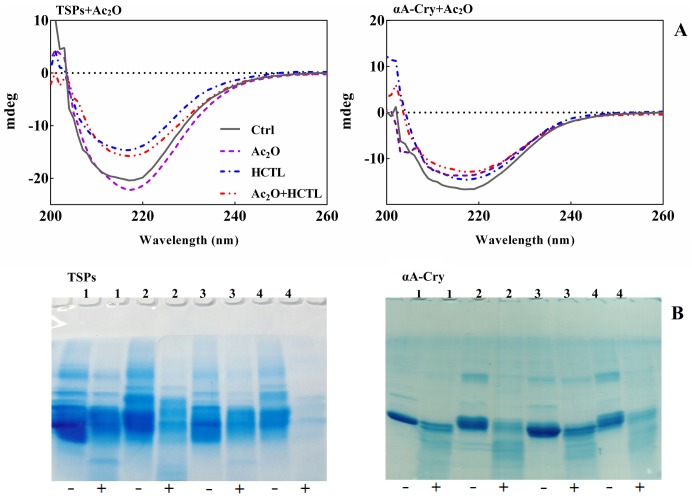
Estimation of the secondary structural contents and evaluation of α-chymotrypsin digestion of lens crystallins upon different modifications. (**A**) CD spectroscopic assessment of lens proteins after modification with Ac_2_O and HCTL. Far UV-CD spectra of TSPs and αA-Cry upon modification with Ac_2_O and HCTL. Proteins were diluted to 0.3 mg mL^-1^ in buffer C. The measurements were undertaken using a cuvette with 0.1 cm path length at 25°C. (**B**) SDS-PAGE assessment of chymotryptic digestion of modified lens proteins. The lens proteins, after modification with Ac_2_O and HTCL, were subjected to reducing SDS-PAGE (12% gel) for the assessment of their proteolytic susceptibility with α-chymotrypsin. The lens proteins (2 mg mL^-1^) were incubated with α-chymotrypsin with a 1:50 (w/w) ratio of enzyme/substrate at 37°C for 6 h. Incubation was done in 100 mM NaPi buffer containing 0.01% NaN_3_ at pH 7.8. At the end of incubation, a 15 μg of each protein sample was loaded to SDS-PAGE gel. 1–4, respectively stand for the control sample, acetylated proteins, HCTL-modified proteins and double modified proteins. Also (-) and (+) indicate the absence and presence of α-chymotrypsin, respectively. The protein bands were visualized by a CBB staining protocol.

In order to further investigate the structural features of the lens crystallins upon two different types of modification; far UV-CD analysis was applied. As shown in Fig 7A, the lens crystallins displayed a minimum ellipticity at a wavelength around 217 nm which is a signature of β-sheet rich structure. Upon acetylation of the lens proteins a slight reduction in this ellipticity minimum was observed. This observation suggests that acetylation alters the β-sheet content of the lens proteins.

The ellipticity minimum peak at 217 nm was significantly reduced for the lens proteins upon modification with either HCTL or with both Ac_2_O and HCTL. These changes suggest the conversion of β-sheet structures to other secondary structures. Modification of αA-Cry with either Ac_2_O or HCTL and subsequent modification of this protein with Ac_2_O and HCTL led to alteration of its CD profile, suggesting secondary structural changes occur upon different modifications of this protein. Among the different αA-Cry samples, the HCTL-modified protein displays significant structural changes with an increased content of β-sheet structures (Table 2).

**Table 2:** The secondary structure content (%) of native and different modified αA-Cry samples.

αA-Cry	α-helix	β-sheet	Turn	Unordered
**Ctrl**	4.3 ± 0.8	40.8 ± 0.7	28.2 ± 1.4	26.7 ± 0.9
**Ac**_**2**_**O**	3.6 ± 1.4	42.5 ± 1.0	27.4 ± 0.7	26.5 ± 1.6
**HCTL**	3.3 ± 1.2	44.6 ± 0.9	25.7 ± 1.2	24.6 ± 1.1
**Ac**_**2**_**O+HCTL**	3.5 ± 0.9	42.9 ± 0.8	27.1 ± 0.9	26.2 ± 1.3

Previous studies suggested a remarkable association between lens crystallins enzymatic degradation and opacification. In our study, we applied limited proteolysis with the aim to assess the susceptibility of the lens crystallins to α-chymotrypsin as a model protease. This sensitive method may indicate the compactness of proteins and might also be considered as a good indicator in determining the protein's surface target residues which are accessible to this protease.

As shown in Fig 7B, the unmodified lens proteins partially resist proteolysis. The extent of proteolytic degradation was significantly enhanced upon modification of both total soluble lens proteins and αA-Cry with Ac_2_O. Unexpectedly, the double-modified lens proteins (TSPs and αA-crystallin) indicate a remarkable extent of proteolytic instability. Therefore, the subsequent or simultaneous modification of the lens proteins with Ac_2_O and HCTL are potential sources of eye lens opacification.

### Analysis of the oligomeric size distribution of the lens crystallins upon modification with Ac_2_O and HCTL

**Fig 8:**
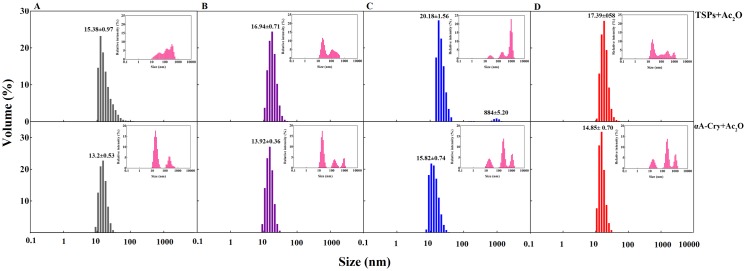
DLS analysis of lens proteins upon modification with Ac_2_O and HCTL. Lens proteins were assessed for their hydrodynamic size distribution, upon modification with Ac_2_O and HCTL, by DLS. Prior to the measurements, the protein samples were diluted to 3 mg mL ^-1^ in 100 mM phosphate buffer, pH 7.4. The size distributions were reported to their relative volumes. The insets indicate their relative scattering intensity. A, B, C and D, respectively indicate native proteins, acetylated proteins, HCTL-modified proteins and double modified proteins.

Dynamic light scattering was applied in order to monitor the aggregation of lens crystallins (TSPs and αA-crystallin) upon modification with Ac_2_O and HCTL (Fig 8). The average size (diameter) of the untreated sample of total lens proteins was 15.38 ± 0.97 nm. Upon acetylation, the average diameter of these proteins slightly increased to 16.94 ± 0.71 nm. Incubation of total soluble lens proteins with HCTL resulted in formation of two populations with the average diameters of 20.18 ± 1.56 nm (91.7%) and 884 ± 5.20 nm (8.3%).

Incubation of the acetylated lens proteins with HCTL led to a shift towards the molecular population with relatively larger size range, i.e. an average diameter of 17.39 ± 0.58 nm. As these experiments were repeated with the untreated and treated samples of αA-Cry, similar results were obtained. Overall, our results suggest that acetylation may prevent the formation of HMW protein species induced by homocysteinylation.

### Assessment of chaperone-like activity of human αA-Cry upon modification with Ac_2_O and HCTL

**Fig 9:**
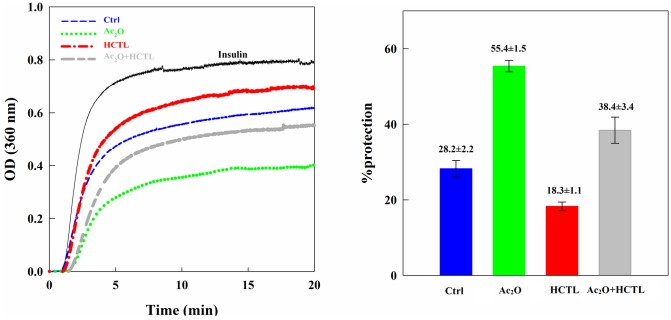
The chaperone-like activity of αA-Cry upon modification with Ac_2_O and HCTL. (**A**) The chaperone-like activity of different modified forms of αA-Cry was assessed in a chemical-induction aggregation system. Native and modified αA-Cry samples (0.15 mg mL^-1^) were assessed to protect the aggregation of bovine pancreatic insulin (0.3 mg mL^-1^) with DTT (20 mM) in NaPi buffer (100 mM) pH 7.2 at 40°C. The aggregation progress was monitored at 360 nm for 20 min. (**B**) The chaperone-like activity was quantified, based on Eq 1, in terms of the percentage of protection.

The chaperone-like activity of αA-Cry was monitored based upon chemical-induction aggregation of the bovine pancreatic insulin (Fig 9). The chaperone-like activity was also expressed as percentage of protection according to Eq 2. Acetylation significantly enhanced the chaperone-like activity of this protein (55.40% ± 1.52% compared to 28.25% ± 2.18% for native αA-Cry). This finding is in agreement with the results of a previous study [23]. Also, similar to our previous report, homocysteinylation markedly reduced the chaperone ability of this protein [10].

Also, the acetylated protein largely preserved the chaperone ability of αA-Cry in the presence of HCTL. Our results suggest that acetylation may have a protective effect on the functional insults induced upon homocysteinylation of αA-Cry.

### Analysis of gel mobility shift and morphological assessment of lens crystallins upon modification with Ac_2_O and HCTL

**Fig 10:**
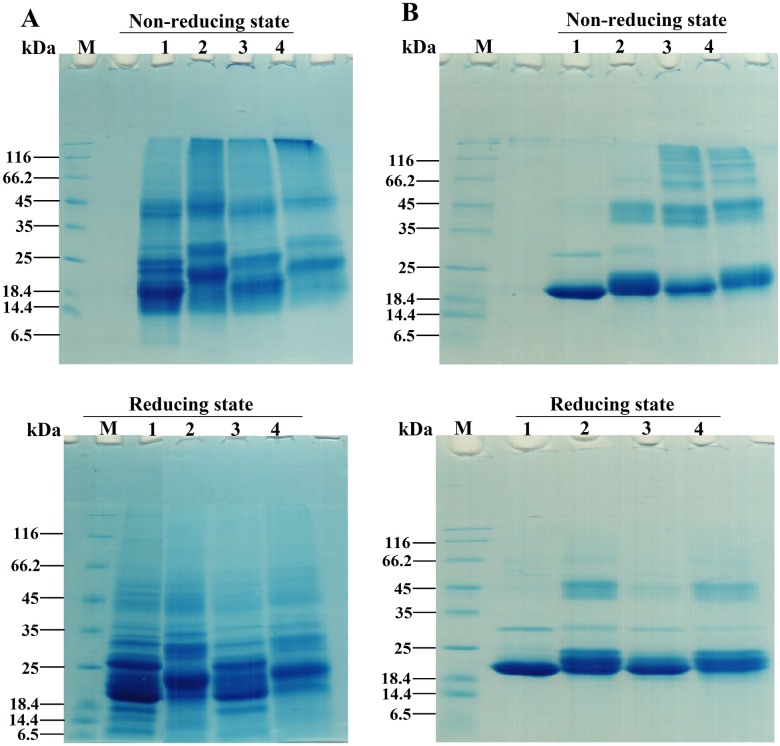
Gel mobility shift analysis of the acetylated lens proteins incubated with HCTL. Native and acetylated TSPs were incubated with HCTL (10 mM) in buffer C at 37°C for 3 days. At the end of incubation, 15 μg of each protein was loaded into wells of electrophoresis gel (12% gel) under both non-reducing and reducing conditions. (**A**) SDS-PAGE analysis of acetylated and double modified TSPs. M indicates the molecular mass markers. Lanes 1–5 respectively are the fresh proteins, control proteins, acetylated proteins, HCTL-modified proteins and HCTL/Ac_2_O double modified proteins. (**B**) SDS-PAGE analysis of acetylated αA-Cry and double modified αA-Cry. Lanes 1–4, respectively are the control αA-Cry, acetylated αA-Cry, HCTL-modified and double modified αA-Cry. The protein bands were visualized by a proper CBB staining method.

Gel mobility shift analysis was performed to investigate the impact of modification with Ac_2_O and HCTL on oligomerization of the lens proteins (Fig 10).

Lanes 1 and 2 are the untreated and acetylated protein samples. Lanes 3 and 4 show their corresponding protein samples which had been incubated with HCTL.

As shown in the non-reducing gels, additional to the increase in mass of the protein bands, acetylation results in formation of HMW protein species (lane 2) in both TSPs and αA-Cry. Applying reducing conditions results in extensive disappearance of HMW protein species, showing significant contribution of disulfide protein crosslinking to the HMW species. However, under reducing condition, the HMW protein species of the acetylated αA-Cry remained largely unaltered, suggesting involvement of other interaction rather than disulfide covalent crosslinking. Incubation of TSPs and αA-Cry (native and acetylated forms) with HCTL results in significant reduction in the intensity of the bands corresponding to the monomeric proteins associated with the appearance of HMW protein species. The HMW protein species largely disappeared in the presence of reducing agent. In the case of the acetylated αA-Cry incubated with HCTL, a protein band corresponding to the dimeric form of this protein was still present under reducing conditions.

**Fig 11:**
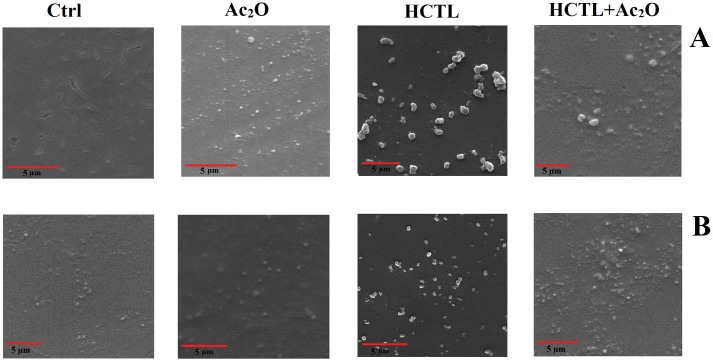
Scanning electron microscope (SEM) images of modified lens crystallins. The symbols are as following: Ctrl: control TSPs; Ac_2_O: acetylated TSPs; HCTL: homocysteineteinylated TSPs and Ac_2_O+HCTL: double-modified TSPs. Also, A and B stand for TSPs and αA-Cry, respectively.

The nature of aggregation in cataractous lenses is a matter of debate but there are two types of well-characterized protein aggregates in cataract lenses: “amorphous” and “amyloid fibrils” [7, 52]. In this study scanning electron microscopy was applied to gain a better understanding of crystallin aggregation upon modification (Fig 11).

As a result of modification with Ac_2_O, an important change was identified in both the morphology and size of the oligomeric assemblies of eye lens crystallins (Fig 11A). After modification with HCTL, the lens proteins displayed a substantial increase in their oligomeric size and significant changes occurred to their morphology. Double modification with both Ac_2_O and HCTL primarily resulted in reduction in the density of the large oligomeric assemblies of the lens proteins with a resultant shift towards lower oligomeric size distributions. Our microscopic observation was also in agreement with the results from DLS analysis (Fig 8). Therefore, acetylation of the lens crystallins may partially prevent the deleterious effects of HCTL modification. As indicated in Fig 11B, acetylation causes a slight increase in αA-Cry aggregation compared to its native counterpart. Also, HCTL-modified αA-Cry displayed increased oligomeric size and aggregated-like assemblies. Double modification of αA-Cry partially restored the oligomeric size of αA-Cry to a similar mass to its native counterpart. Overall, we concluded from our microscopic assessments that the aggregation pathways were significantly different when the lens crystallins were subjected to various types of modification.

## Discussion

Modification of protein lysine residues by glucose is believed to underlie an increased risk of diabetes and Alzheimer’s disease [53, 54]. Also, this amino acid residue is an important target of protein peniciloylation, as detected in penicillin allergy [55, 56]. The involvement of protein lysine residues as a site of modification has also been shown during the deleterious reaction between the oxidation products of lipids and proteins as implicated in the etiology of atherosclerosis [57]. In addition, protein homocysteinylation has been associated with the pathology of various human disorders such as cardiovascular diseases, neurodegenerative illnesses and several ocular ailments [9, 58]. Homocysteinylation of the lens proteins through post-translational acylation of lysine and to a minor extent through the reaction with sulfhydryl group of cysteine has been suggested to be an important underlying cause of homocysteine toxicity in cataract diseases [10, 32]. The previous studies suggested that acetylation of lysine residues in the lens proteins can prevent their non-enzymatic glycation, carbamylation and inhibits their reaction with cyanate; hence reducing human cataract development [24, 28, 29]. Recent investigations indicate that acetylation of the lens crystallins occurs very early and continues throughout life and does not exhibit striking detrimental effects on these proteins [23].

Our study was conducted to assess the potential capacity of acetylation as a new approach for possible inhibition of the adverse effects of homocysteinylation on the structure and function of lens crystallins. From our results, acetylation demonstrates a partial shielding effect against the structural changes induced upon lens proteins homocysteinylation and significantly prevents their aggregation in the presence of HCTL (Fig 5). Also, acetylation effectively decreased the average diameter enhancement of these proteins occurring by homocysteinylation (Fig 8). Structural integrity is highly significant for the proper and fine interactions between the different crystallin subunits, playing important role in maintenance of the lens transparency. In addition, formation of protein aggregates is an important event in various human pathologies including cataract. In agreement with the previous report [23], our results also suggest that acetylation improves chaperone-like activity of α-Cry. In addition, acetylation partially restored the chaperone-like activity of this protein which is attenuated in the presence of HCTL (Fig 9). Comparing the results of ANS fluorescence (Fig 6) and chaperone activity assessment (Fig 9), indicates that there is a direct correlation between the extent of hydrophobicity and chaperone activity of αA-Cry upon acetylation.

**Fig 12:**
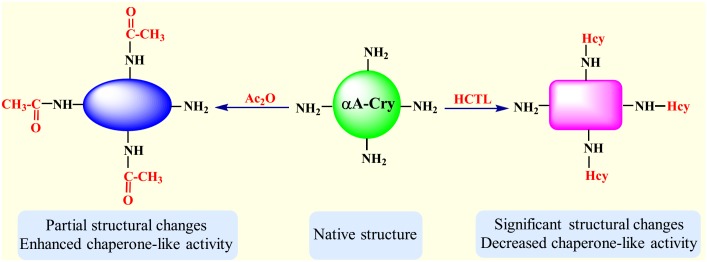
Structural properties and chaperone-like activity of αA-Cry upon modification with Ac_2_O and HCTL. Homocysteinylation of free amino groups results in significant structural changes and attenuates the chaperone-like activity of αA-Cry while acetylation leads to partial structural alteration and enhanced efficiency in suppressing aggregation of a client protein.

The free amino groups especially the epsilon amino group of lysine residues in proteins, are the primary target of both acetylation and homocysteinylation [18, 19]. It seems that the degree of structural alteration of αA-Cry is an important determining factor in its chaperoning action. While modification of amino groups with HCTL exerts significant structural changes and reduces the chaperone ability of αA-Cry, acetylation of these residues with Ac_2_O results in partial structural alteration which is accompanied by enhanced chaperone-like activity (Fig 12).

Acetylation also partially prevents the significant increase in sulfhydryl contents of these proteins after modification with HCTL (Fig 4A). As shown in Fig 10, lens crystallins homocysteinylation leads to incorporation of additional sulfhydryl groups into protein structure which facilitates protein crosslinking via formation of Disulfide Bridge. Also, as mentioned previously, disulfide bond protein crosslinking can facilitate protein aggregation which is a significant risk factor in cataract development [10, 32, 59, 60]. Despite of its beneficial affects against structural and functional insults induced by HCTL, acetylation was also increased the proteolytic instability of lens proteins (Fig 7B). In the lenticular tissues, unexpected activity of a calcium-dependent protease (calpain) results in degradation and subsequently insolubilzation of crystallins, leading to lens opacity [61]. Acetylation caused a shielding effect against the structural and functional damages induced by HCTL in the lens crystallins but increased proteolytic instability of the lens proteins upon this modification is a major drawback. As mentioned previously, during hyperhomocysteinemia lens crystallins are potential targets of homocysteinylation which has been also indicated in the pathogenesis of cataract development. Therefore, further investigation is needed for better analysis of the role of acetylation as a novel preventive approach against homocysteinylation-associated structural and functional damage to lens crystallins, particularly in patients with hyperhomocysteinemia.
